# The anti‐inflammatory and antiapoptotic effects of probiotic on induced neurotoxicity in juvenile hamsters

**DOI:** 10.1002/fsn3.2435

**Published:** 2021-07-13

**Authors:** Abir Ben Bacha, Norah Al‐Orf, Mona Alonazi, Ramesa Shafi Bhat, Afaf El‐Ansary

**Affiliations:** ^1^ Biochemistry Department Science College King Saud University Riyadh Saudi Arabia; ^2^ Laboratory of Plant Biotechnology Applied to Crop Improvement Faculty of Science of Sfax University of Sfax Sfax Tunisia; ^3^ Central Laboratory King Saud University Riyadh Saudi Arabia

**Keywords:** apoptosis, clindamycin, cytokines, heat shock protein, propionic acid

## Abstract

Brain inflammation and apoptosis play crucial roles in the pathogenesis of various neurodevelopmental disorders. Probiotics have been shown to confer protection against many stresses, including apoptosis and inflammation, by modulating the gut function. The short‐chain fatty acid, propionic acid (PPA), plays an important intermediate of cellular metabolism. Although PPA exhibits numerous beneficial biological effects, its accumulation is neurotoxic. This study focused on the therapeutic potency of probiotics against PPA‐induced apoptosis and neuroinflammation in hamsters. Five groups of male golden Syrian hamsters were treated as follows: Group I as control; Group II as PPA‐treated with three doses of 250 mg PPA/kg/day; Group III as clindamycin‐treated with a single dose of 30 mg clindamycin/kg; Group IV as PPA–probiotic; and Group V as clindamycin–probiotic were two therapeutic groups which were treated with the same doses of PPA and clindamycin, respectively, followed by treatment with 0.2 g kg^‐1^ d^−1^ of probiotic (Protexin^R^, Probiotics International Limited) for three weeks. Proapoptotic markers, such as caspases 3 and 7; neuroinflammation markers, such as interleukins 1β and 8; and heat shock protein 70 were measured in the brain. Significant increase of all measured markers (*p* ˂ .001) was observed in PPA and clindamycin‐treated hamsters compared with controls. Probiotics significantly reduced the damages and ameliorated all the test markers in both therapeutic groups compared with the control. Our results confirmed that probiotics can be utilized as a feasible strategy for managing apoptotic and inflammation‐related stresses in brain disorders by retaining the gut function.

## Graphical abstract

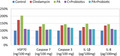

## 1 INTRODUCTION

In recent years, the gut microbiota has become the subject of extensive research as alterations in gut microbiota composition have been found to influence host physiology and seem to be linked with a number of diseases, including neurological disorders, such as schizophrenia, autism, anxiety disorders, and major depressive disorders (Inserra et al., 2018).

Increased emphasis has been placed on the role of gut microbiota during the development of the nervous system (Borre et al., 2014); bidirectional communication between the central nervous system (CNS) and microbiota is essential for monitoring and integrating intestinal functions as well as linking them with the emotional and cognitive centers of the brain, and plays important roles in mechanisms such as immune activation, intestinal permeability, enteric reflex, and enteroendocrine signaling. Moreover, the bidirectional communication between the CNS and gut microbiota maintains homeostasis and may affect pain perception, stress reactivity, neurochemistry, and various brain–gut axis disorders (Collins et al., 2012; Cryan & Dinan, 2012; Foster, 2013).

Propionic acid (PPA) is an environmental factor that causes neuroinflammation and various behavioral changes, which may be linked with the development of neurodevelopmental disorders such as autism (El‐Ansary et al., 2012). Propionic acid and other short‐chain fatty acid (SCFAs) are synthetized through the degradation of dietary amino acids and carbohydrates by many intestinal bacteria, including those involved in antibiotic‐associated diarrhea (Kuijper et al., 2006) such as *Clostridia*. Generally, SCFAs are metabolized in the liver (Thomas et al., 2012); however, due to acquired and/or genetic metabolic abnormalities (MacFabe et al., 2007; Wajner et al., 2004), high blood levels of SCFAs can accumulate and gain access to the CNS after crossing the gut–blood and blood–brain barriers (BBB). Consequently, intracellular acidification may occur (Bonnet et al., 2000; Karuri et al., 1993), which affects cell signaling (Nakao et al., 1998), immune function (Le Poul et al., 2003), neurotransmitter release (DeCastro et al., 2005), lipid metabolism (Hara et al., 1999), mitochondrial function/CoA sequestration (Wajner et al., 2004), gene expression (Parab et al., 2007), and gap junction modulation (Rörig et al., 1996), thus causing neuropathologic and biochemical abnormalities. Numerous studies indicated correlation between behavioral abnormalities and neuroinflammation in rats exposed to PPA (El‐Ansary et al., 2012; Shultz et al., 2008). Therefore, PPA seems to affect both the brain and behavior of laboratory animals in a way that is consistent with symptoms of neurological disorders. Intracerebroventricular (ICV) injection or oral administration of neurotoxic doses of PPA efficiently induced hyperactivity, repetitive behaviors, turning behavior, kindled seizures, retropulsion, neuroinflammation, and widespread oxidative stress in rats (MacFabe et al., 2007).

Moreover, animal models of neurotoxicity can be established through different methods, including the use of clindamycin, a broad‐spectrum antibiotic prescribed for the treatment of bacterial infections (von Konow et al., 1992). Clindamycin has been employed to favor the overgrowth of propionic acid‐producing bacteria such as *Clostridium* sp. and indirectly elevates PPA levels. Similarly to other antibiotics, clindamycin affects both pathogen and commensal host microbiota, thus inducing intestinal dysbiosis and stimulating the gut microbiota associated with immunologic, metabolic, and developmental disorders, besides increasing the susceptibility to infectious diseases (Cuthbertson et al., 2016; El‐Ansary et al., ,^2013^, ^2018^; Holmes et al., 2008). Buffie et al. (2012) and Al‐Orf et al. (2018) revealed that a single dose of clindamycin could significantly decrease the diversity of intestinal microbiota, favor the growth of *Clostridia,* and promote the development of colitis and diarrhea.

Furthermore, the microbiota–gut–brain axis concept opens new horizons for developing novel therapies based on the modulation of gut microbiota to prevent or alleviate neurodevelopmental disorders. Therefore, consumption of fermented foods, which contain various probiotic strains, could play a major role in the therapy of several neurodevelopmental disorders, including anxiety disorder, autism, Parkinson's disease, and multiple sclerosis (Fung et al., 2017). Microorganisms that act as probiotics have different properties; they persistently adhere to intestinal epithelial cells and mucus, reduce and exclude pathogenic adherence to healthy cells, propagate in a manner that allows fast multiplication and colonization, and generate reactive agents such as bacteriocins, hydrogen peroxide, and acids that can hamper pathogen multiplication. In addition, probiotics are safe, nonpathogenic, noncarcinogenic, and resist several microbicides, allowing them to form a balanced flora (Reid et al., 1990).

Probiotics exhibit numerous potential therapeutic effects against human diseases (including lactose maldigestion, diarrhea, inflammatory bowel disease, colorectal cancer, immune diseases, gut disorders, autism, constipation, colitis, pathogen colonization, flatulence, gastroenteritis, gastric acidity, hypercholesterolemia, hepatic encephalopathy, and carcinogenesis); however, few studies explored the beneficial effects of probiotics on neuroinflammation. For this reason, the present study evaluated the impact of probiotics on certain proapoptotic caspases [Caspases (CASP) 3 and 7], heat shock protein 70 (HSP70), and neuroinflammatory factors [Interleukin (IL‐) 1β and 8] in the hamster brain as complementary study to our most recent published works (Al‐Orf et al., 2018; El‐Ansary et al., 2018).

## 2 MATERIALS AND METHODS

### 2.1 Animals

All animal experiments were approved by the Ethics Committee of the College of Science at King Saud University and were carried out according to the national guidelines for use and care of animals.

Fifty young male golden Syrian hamsters (64.5 ± 3.9 g, 21 days old) were randomly assigned to five groups of ten hamsters each. Animals in the control group were administered phosphate‐buffered saline (PBS) via an orogastric tube daily. Hamsters in the PPA (Sigma‐Aldrich)‐treated group were orally administered 250 mg PPA/kg/day for three consecutive days, followed by PBS till the end of experiment duration, whereas those in the clindamycin (Azupharma—Germany)‐treated group received a single dose of clindamycin (30 mg/kg) through an orogastric tube followed by PBS, and were sacrificed the following day (El‐Ansary et al., 2012). Animals in the two therapeutic groups were administered the same doses of PPA or clindamycin, followed by treatment with 0.2 g probiotic/kg/day dissolved in phosphate‐buffered saline for three weeks. The probiotic used was Protexin^R^, (Probiotics International Limited, Somerset, UK), which consists of a combination of *Bifidobacterium brev*e, *Bifidobacterium infantis*, *Lactobacillus acidophilus*, *Lactobacusus bulgar*, *Lactobacillus casei*, *Lactobacillus rhamnosus*, and *Streptococcus thermophilus* 1 109 CFU). Hamsters were maintained at 21 ± 1℃ on a standard diet and water ad libitum.

Carbon dioxide‐anesthetized hamsters were decapitated at the end of feeding trials, and their brains were removed from the skull and dissected into small pieces. After homogenization in bidistilled water (1:10, w/v), brain samples were stored at −80°C.

### 2.2 Biochemical analyses

CASP 3 (Ref. MBS1602954), CASP 7 (Ref. MBS761582), IL‐ 1β (Ref. MBS7700778), IL‐ 8 (Ref. MBS025179), and HSP70 (Ref. MBS035085) were investigated in the brain homogenates using ELISA kits (MyBioSource) following the manufacturer's instructions. All measurements were performed in duplicate, and the mean of two different readings was calculated. Quality control assays were performed to evaluate experimental reproducibility through the inter‐ and intra‐assay coefficients of variability (%CV).

### 2.3 Statistical analysis

Data were analyzed using the statistical package for the social sciences (SPSS). Results are presented as mean and standard deviation (*SD*). Statistical comparisons and correlations between parameters were performed using the independent *t* test and Pearson's correlation coefficient (*r*), respectively. Furthermore, the receiver operating characteristics (ROC) curve and the area under the ROC curve (AUC) were used as a fundamental tool to evaluate brain neurotoxicity in animal modeling. Only *p* values ≤.005 were considered significant.

## 3 RESULTS

**FIGURE 1:**
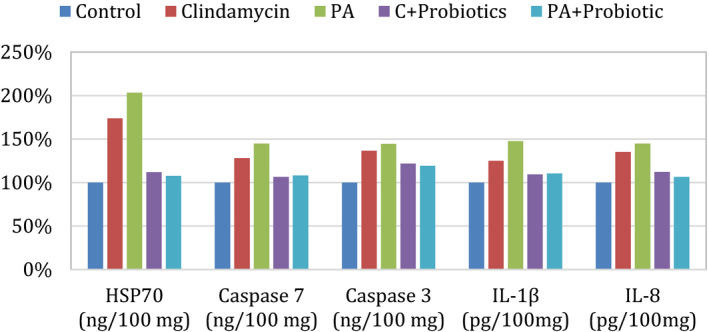
Percentage change for all parameters in all groups compared with the control

The data of the current study are expressed as mean ± *SD* of all investigated biochemical parameters in the different studied groups. Levels of CASP 3 and 7, HSP70, and IL‐8 and IL‐1 in the brain homogenates of the 5 groups as well as their percentage change in the treatment groups compared with the control group are reported in Table 1 and Figure 1.

**TABLE 1:** Mean ± *SD* of all measured parameters in the brain homogenates of the 5 groups of hamsters studied (*p* < .05)

Parameters	Groups	*N*	Min.	Max.	Mean ± *SD*	*p* value^a^	*p* value^b^
HSP70 (ng/100 mg)	Control	8	24.70	30.10	27.50 ± 1.80		>.001
	Clindamycin	8	41.00	55.00	47.75 ± 4.68	>.001	
	PPA	8	47.00	62.00	55.88 ± 5.03	>.001	
	Clindamycin + Probiotics	8	27.00	36.00	30.75 ± 2.92	.421	
	PPA + Probiotics	8	15.00	36.00	29.63 ± 6.61	.755	
CASP 7 (ng/100 mg)	Control	8	105.00	121.00	112.50 ± 5.76		>.001
	Clindamycin	8	132.00	155.00	144.25 ± 7.38	>.001	
	PPA	8	150.00	176.00	163.00 ± 9.09	>.001	
	Clindamycin + Probiotics	8	110.00	126.00	119.88 ± 5.64	.167	
	PPA + Probiotics	8	109.00	135.00	121.63 ± 8.63	.063	
CASP 3 (ng/100 mg)	Control	8	88.00	102.00	94.53 ± 5.02		>.001
	Clindamycin	8	118.00	139.00	129.00 ± 7.58	>.001	
	PPA	8	125.00	145.00	136.50 ± 6.99	>.001	
	Clindamycin + Probiotics	8	105.00	127.00	115.13 ± 7.41	>.001	
	PPA + Probiotics	8	103.00	125.00	112.75 ± 7.61	>.001	
IL‐1β (pg/100 mg)	Control	8	102.00	125.00	112.38 ± 8.50		>.001
	Clindamycin	8	126.00	150.00	140.50 ± 8.40	>.001	
	PPA	8	155.00	173.00	165.88 ± 6.96	>.001	
	Clindamycin + Probiotics	8	109.00	138.00	122.88 ± 9.14	.062	
	PPA + Probiotics	8	109.00	136.00	124.25 ± 9.41	.029	
IL‐8 (pg/100 mg)	Control	8	107.00	135.00	120.25 ± 9.60		>.001
	Clindamycin	8	154.00	174.00	162.50 ± 6.39	>.001	
	PPA	8	160.00	183.00	174.00 ± 6.93	>.001	
	Clindamycin + Probiotics	8	121.00	145.00	134.75 ± 7.65	.009	
	PPA + Probiotics	8	112.00	146.00	128.13 ± 12.56	.248	

^a^ *p* value between each group and the control group.

^b^ *p* value between all groups.

Table 1 and Figure 1 clearly show increased levels of proapoptotic markers CASP 3 and 7, and stress‐induced protein HSP70 together with a remarkable increase in the production of proinflammatory cytokines IL‐8 and IL‐1β in the brain homogenates of PPA‐ and clindamycin‐treated hamsters compared with the control group.

Table 2 demonstrates Pearson's correlation coefficients showing positive and negative correlation between the measured parameters while Table 3 shows the multiple regressions of the measured variables. It can be easily observed that HSP70 as a dependent variable could be greatly affected by CASP 7 and IL‐8 as predictive variables (*R* ^2^ of .812).

**TABLE 2:** Pearson's correlation between all tested parameters

Parameters	*R* (Spearman Correlation)	*p* value	
HSP70 ~ CASP 7	.880**	>.001	P^a^
HSP70 ~ CASP 3	.792**	>.001	P^a^
HSP70 ~ IL−1β	.839**	>.001	P^a^
HSP70 ~ IL−8	.868**	>.001	P^a^
CASP 7 ~ CASP 3	.787**	>.001	P^a^
CASP 7 ~ IL−1β	.851**	>.001	P^a^
CASP 7 ~ IL−8	.859**	>.001	P^a^
CASP 3 ~ IL−1β	.803**	>.001	P^a^
CASP 3 ~IL−8	.803**	>.001	P^a^
IL−1β ~ IL−8	.812**	>.001	P^a^

^a^ Positive correlation.

** Correlation is significant at the .01 level.

**TABLE 3:** Multiple Regression using Stepwise method between the measured markers, HSP70, CASP 7, CASP 3, IL‐1β, and IL‐8

Predictor Variable	Coefficient	*SE*	*p* value	Adjusted *R* ^2^	95% CI	
					Lower	Upper
CASP 7 (ng/100 mg)	0.536	0.047	≤.01	.768	0.441	0.631
CASP 7 (ng/100 mg)	0.312	0.082	≤.01	.812	0.145	0.479
IL−8 (pg/100mg)	0.232	0.074	.001		0.083	0.381
HSP70 (ng/100 mg)	1.445	0.127	≤.01	.768	1.188	1.701
HSP70 (ng/100 mg)	0.919	0.213	≤.01	.807	0.488	1.350
IL−1β (pg/100 mg)	0.375	0.127	≤.01		0.117	0.633
IL−8 (pg/100 mg)	0.572	0.069	≤.01	.635	0.432	0.711
IL−8 (pg/100 mg)	0.316	0.108	.011	.695	0.097	0.534
IL−1β (pg/100 mg)	0.347	0.119	.006		0.106	0.587
CASP 7 (ng/100 mg)	0.866	0.087	≤.01	.717	0.691	1.042
CASP 7 (ng/100 mg)	0.587	0.130	.794	.758	0.324	0.850
CASP 3 (ng/100 mg)	0.445	0.163	≤.01		0.115	0.775
HSP70 (ng/100 mg)	1.595	0.148	≤.01	.746	1.295	1.896
HSP70 (ng/100 mg)	0.912	0.289	.003	.782	0.326	1.498
CASP 7 (ng/100 mg)	0.473	0.176	.003		0.116	0.830

Table 4 shows the ROC curves of the measured variables. It can be easily noticed that the 5 parameters recorded high AUCs, with satisfactory specificity and sensitivity.

**TABLE 4:** ROC analysis of all measured parameters in all groups

Parameters	Groups	AUC	Cutoff value	Sensitivity %	Specificity %	*p* value	95% CI
HSP70 (ng/100 mg)	Clindamycin	1.000	35.550	100.0%	100.0%	.001	1.000–1.000
	PPA	1.000	38.550	100.0%	100.0%	.001	1.000–1.000
	Clindamycin + Probiotics	0.820	28.750	75.0%	75.0%	.031	0.611–1.030
	PPA + Probiotic	0.758	28.750	75.0%	75.0%	.083	0.497–1.019
CASP 7 (ng/100 mg)	Clindamycin	1.000	126.500	100.0%	100.0%	.001	1.000–1.000
	PPA	1.000	135.500	100.0%	100.0%	.001	1.000–1.000
	Clindamycin + Probiotics	0.828	114.000	87.5%	62.5%	.027	0.627–1.030
	PPA + Probiotic	0.812	116.500	75.0%	75.0%	.036	0.599–1.026
CASP 3 (ng/100 mg)	Clindamycin	1.000	110.000	100.0%	100.0%	.001	1.000–1.000
	PPA	1.000	113.500	100.0%	100.0%	.001	1.000–1.000
	Clindamycin + Probiotics	1.000	103.500	100.0%	100.0%	.001	1.000–1.000
	PPA + Probiotic	1.000	102.500	100.0%	100.0%	.001	1.000–1.000
IL‐1β (pg/100 mg)	Clindamycin	1.000	125.500	100.0%	100.0%	.001	1.000–1.000
	PPA	1.000	140.000	100.0%	100.0%	.001	1.000–1.000
	Clindamycin + Probiotics	0.805	114.000	87.5%	62.5%	.041	0.589–1.020
	PPA + Probiotic	0.844	121.500	75.0%	87.5%	.021	0.650–1.038
IL‐8 (pg/100 mg)	Clindamycin	1.000	144.500	100.0%	100.0%	.001	1.000–1.000
	PPA	1.000	147.500	100.0%	100.0%	.001	1.000–1.000
	Clindamycin + Probiotics	0.883	126.500	87.5%	75.0%	.010	0.715–1.050
	PPA + Probiotic	0.695	129.500	50.0%	87.5%	.189	0.430–0.961

## 4 DISCUSSION

Probiotics are live microorganisms that display health‐promoting effects, prevent pathogen colonization of the gut, and ameliorate the symptoms of numerous diseases caused by deregulations of the immune system. Considering that the beneficial effects of probiotics can vary between strains, identifying the most efficient probiotic will be critical for their use in the prevention or treatment of various diseases. The present study explored the therapeutic effects of a combination of probiotics.

Table 1 shows increased levels of proapoptotic markers CASP 3 and 7, and stress‐induced protein HSP70 in the brain homogenates of PPA‐ and clindamycin‐treated hamsters compared with the control group. The proapoptotic effects of PPA corroborate with the recent report of Lobzhanidze et al. (2019) who conclude that despite the beneficial activity of PPA as mediator between the gut microbiota, nutrition, and brain physiology, excessive levels of this SCFA can cause detrimental effects, including oxidative stress, mitochondrial dysfunction, developmental delay, and immune and metabolic alterations (Chapman et al., 2015; MacFabe et al., 2007). Additionally, PPA readily crosses the gut–brain barrier (GBB), causes neurochemical changes, alters brain signaling, and induces mitochondrial dysfunction‐related apoptosis (De Almeida et al., 2006; El‐Ansary et al., 2018; MacFabe, 2013; MacFabe et al., 2007; Xu et al., 2016). In rodent model of autism, low doses of PPA were found to significantly decrease social interaction, which could be attributed to significant modifications in the structure/ultrastructure of the amygdala. The marked increase in CASP 3 levels as a neurotoxic event is in good agreement with the previous study of Khalil et al. (2015) who reported the upregulation of CASP 3 mRNA expression in the brain of PPA‐treated rat pups, concomitant with the downregulation of Bcl‐2, an antiapoptotic protein. Elevated Bax/Bcl‐2 ratio is a potential molecular marker of increased apoptosis and can be caused by the loss of Bcl‐2 protein (Khodapasand et al., 2015). Increased values of this ratio correlate with increased mitochondrial membrane permeability after Ca^2+^ release from the endoplasmic reticulum in response to oxidative stress, another neurotoxic effect of PPA. This process culminates with the release of cytochrome c (Cytc), a crucial factor in the caspase‐dependent cell death pathway (Acehan et al., 2002; El‐Ansary et al., 2012; Nutt et al., 2002).

Heat shock proteins (HSPs) are a group of extremely conserved proteins that are usually identified as intracellular chaperones (Rudiger et al., 1997). The recorded increase in HSP70 levels is in good agreement with the previous work of Stao et al. (1997) who reported that BBB disruption, an event preceding neurotoxicity, is often followed by increased HSP70 expression and apoptosis. This could also be related to our earlier study in which oral administration of PPA induced a significant increase in tail moment (comet DNA assay), tail length, and DNA fragmentation in PPA‐treated rats compared with the control (El‐Ansary et al., 2012).

Upon exposure to stress, such as infection and inflammation, HSP70 is released through unidentified mechanisms into the body fluids where it acts as an endogenous damage‐associated molecular pattern to alter the secretion of proinflammatory cytokines (Ellis, 1990; Young, 1990). This concept is also supported by data showing that significantly elevated HSP70 levels were accompanied by a remarkable increase in the production of proinflammatory cytokines IL‐8 and IL‐1β. It is well accepted that HSP70 plays a key role in inflammation by binding to toll‐like receptor TLR4, thus activating nuclear factor kappa B (NF‐κB) signaling to stimulate the production of inflammatory cytokines (Asea et al., 2000; Chalmin et al., 2010; Lee, et al., 2019; Lee, Jeon, et al., 2019).

Numerous probiotics, including *Bifidobacterium, Lactobacillus*, and *Bacillus,* are commonly used in therapy due to their beneficial effect on growth promotion and disease suppression (Král et al., 2012).

The antiapoptotic effects of the chosen probiotics (combination of *Bifidobacterium brev*e, *Bifidobacterium infantis*, *Lactobacillus acidophilus*, *Lactobacusus bulgar*, *Lactobacillus casei*, *Lactobacillus rhamnosus*, and *Streptococcus thermophilus*) can be explained on the basis of higher Bax/Bcl‐2 ratio, which accelerated the formation of Cytochrome c/Apaf1/CASP 9 protein–polymer combinations, followed by the cleavage and activation of CASP 3, eventually leading to cell apoptosis and tissue damage (Salakou et al., 2007). More recently, Wu et al. (2019) demonstrated that probiotics downregulated Bax while upregulating Bcl‐2 in the ileum mucosa.

Among the probiotics investigated in the present study, *Bifidobacterium* and *Lactobacillus* are the most commonly used to treat gut dysbiosis. They are supposed to restore the composition of gut microbiota by promoting healthy bacterial growth through immunomodulatory action in gut (Lee, Lee, et al., 2019; Lee, Jeon, et al., 2019; Schiffrin et al., 2009; Xia et al., 2011; Zhang et al., 2010). Bacterial cells interact with a wide variety of cells that are present in the intestine, such as epithelial cells, dendritic cells, and macrophages, which further stimulate the secretion of pro‐ and anti‐inflammatory cytokines (Chon & Choi, 2010; Rodes et al., 2013; Roselli et al., 2006; Zhang et al., 2019). Certainly, these inflammation‐related markers can affect the brain via the bidirectional gut–brain axis.

Although numerous publications in this field have emphasized the ability of probiotics to decrease local intestinal inflammation, it is also well established that probiotics have peripheral anti‐inflammatory potency beyond the gastrointestinal tract. For instance, studies in both animal models and humans have recorded decreased levels of systemic proinflammatory cytokines following probiotics ingestion (i.e., IL‐1β, IL‐8, TNF‐α, IL‐6, and IFN‐γ; Schachter et al., 2018).

Much attention has been focused on clarifying the pathways through which inflammatory signals could diffuse from the periphery to the brain (D’Mello et al., 2009; Miller et al., 2009; Quan & Banks, 2007). Although these pathways still remain to be fully elucidated, some reports have hinted at the mechanism of direct diffusion of peripheral proinflammatory cytokines into the brain by binding to saturable transport proteins. Owing to their relatively large size, saturable transport receptors typically bind circulating proinflammatory cytokines and transport them into the brain across the BBB (Quan & Banks, 2007). However, direct entry of peripheral inflammatory cytokines into the brain through leaky regions of the BBB as a consequence to the neurotoxic effects of PPA has also been reported (El‐Ansary et al., 2012; Quan & Banks, 2007).

Table 2 demonstrates the high significant positive correlation between the five measured variables. This was understood considering the linear multiple regressions. Table 3 demonstrates the multiple regressions of the measured variables. It can be easily observed that HSP70 as a dependent variable could be greatly affected by CASP 7 and IL‐8 as predictive variables (*R* ^2^ of .812). This corroborates with previous results supporting the link between HSP70 and apoptosis (Stao et al., 1997), NF‐κB activation, and inflammatory cytokine production. This relationship can be confirmed by data presented in the same table, which shows the contribution of HSP70 and IL‐8 as independent variables in the variations of CASP 7 (*R* ^2^ of .807), and both measured cytokines in the alteration of CASP 3 levels (*R* ^2^ of .695). As expected, the proinflammatory molecules IL‐8 and IL‐1β as dependent variables were found to be affected with variable degrees by HSP70 and caspases 3 and 7 as proapoptotic‐related parameters.

Table 4 shows the ROC curves of the measured variables. It can be easily noticed that the 5 parameters recorded high AUCs, with satisfactory specificity and sensitivity.

## 5 CONCLUSION

This study ascertained the role of neuroinflammation and apoptosis as neurotoxic effects in the PPA‐induced rodent model of autism. Clindamycin showed relatively fewer neurotoxic effects compared with orally administered PPA. Moreover, our study showed that probiotics exert ameliorative effects and may be used as a novel noninvasive and safe treatment strategy.

## CONFLICT OF INTEREST

The authors declare no potential conflicts of interest with respect to the authorship, and/or publication of this article.

## AUTHOR CONTRIBUTIONS

**Abir Ben Bacha:** Conceptualization (equal); Data curation (equal); Supervision (equal); Writing‐review & editing (equal). **Norah Al‐Orf** **:** Investigation (equal); Methodology (equal); Writing‐original draft (equal). **Mona Alonazi:** Data curation (equal); Funding acquisition (equal); Project administration (equal). **Ramesa Shafi Bhat:** Formal analysis (equal); Methodology (equal); Software (equal). **El‐Ansary A:** Supervision (equal); Validation (equal); Writing‐review & editing (equal).

## ETHICAL APPROVAL

All procedures performed in studies involving animals were in accordance with the ethical standards of the institution or practice at the Faculty of King Saud University (KSU‐SE‐17–10).

## CONSENT TO PARTICIPATE

Not applicable.

## CONSENT TO PUBLISH

Not applicable.

## Data Availability

The data used to support the findings of this study are available from the corresponding author upon request.
